# Supplementary material for: On the benefits of the tryptophan metabolite 3-hydroxyanthranilic acid in Caenorhabditis elegans and mouse aging
Source: Nat Commun. 2023 Dec 14;14:8338. doi: 10.1038/s41467-023-43527-1 (PMC10721613; doi:10.1038/s41467-023-43527-1)
Supplement: Supplementary file 1 — Supplementary Information [file 41467_2023_43527_MOESM1_ESM.pdf]

SUPPLEMENTARY INFORMATION

On the benefits of the tryptophan metabolite 3-hydroxyanthranilic acid in *Caenorhabditis elegans* and mouse aging

Supplementary Figures

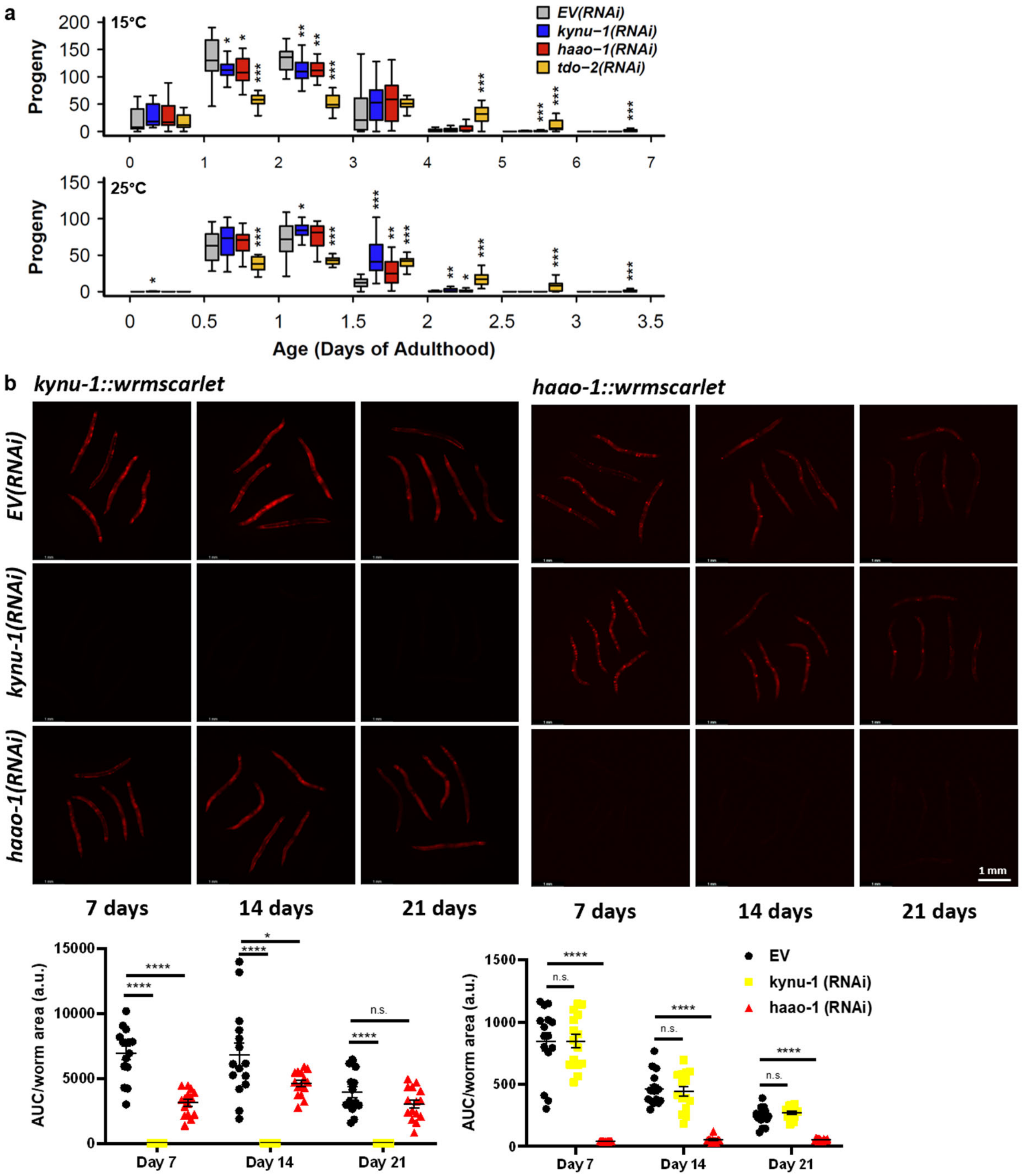

Supplementary Figure 1. Knockdown of *kynu-1*, *haao-1*, or *tdo-2* delays egg production and alters kynurenine pathway metabolic activity. RNAi knockdown of *kynu-1*, *haao-1*, or *tdo-2* (**a**) results in delayed egg production relative to *EV(RNAi)*. (**b**) *kynu-1(RNAi) and haao-1(RNAi)* efficiently knockdown expression of transgenic fluorescent fusion reports *kynu-1::wrmscarlet* and *haao-1::wrmscarlet*, respectively. Fluorescent panels (top) show representative images of the indicated worms (5 worms/panel). Charts (bottom) show quantified fluorescence intensity for individual animals in each group. Error bars indicate standard error of mean. * $p < 0.05$, ** $p < 0.01$, *** $p < 0.001$ compared to age-matched *EV(RNAi),* wild type, or 0 mM control (Welch's t test, panels a, b). Summary statistics are provided in Supplementary Data 1 and source data are provided in Supplementary Data 2.

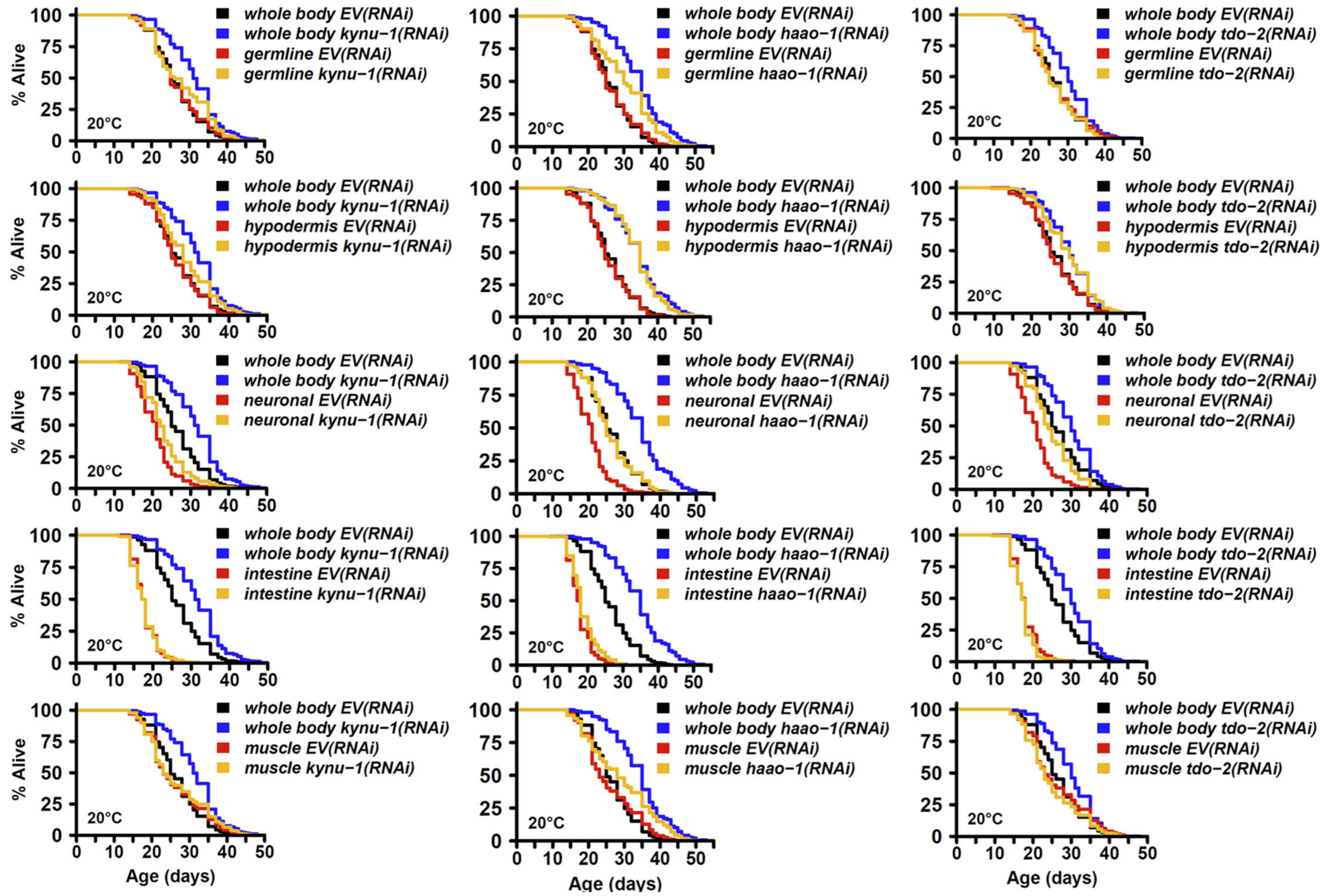

Supplementary Figure 2. The impact of *kynu-1*, *haao-1*, and *tdo-2* knockdown on lifespan show distinct patterns of tissue dependence. RNAi knockdown *tdo-2*, *kynu-1*, or *haao-1* in wild type animals compared to mutants or transgenic animals modified such that RNAi is only effective in specific tissues: germline (strain MAH23), hypodermis (strain NR222), neurons (strain TU3335), intestine (strain VP303), or muscle (strain WM118). Each panel shows the impact on *C. elegans* survival for the indicated kynurenine pathway RNAi vs. *EV(RNAi)* in wild type (N2) animals and one strain with active RNAi only in the indicated tissue. Summary statistics are provided in Supplementary Data 1 and source data are provided in Supplementary Data 2.

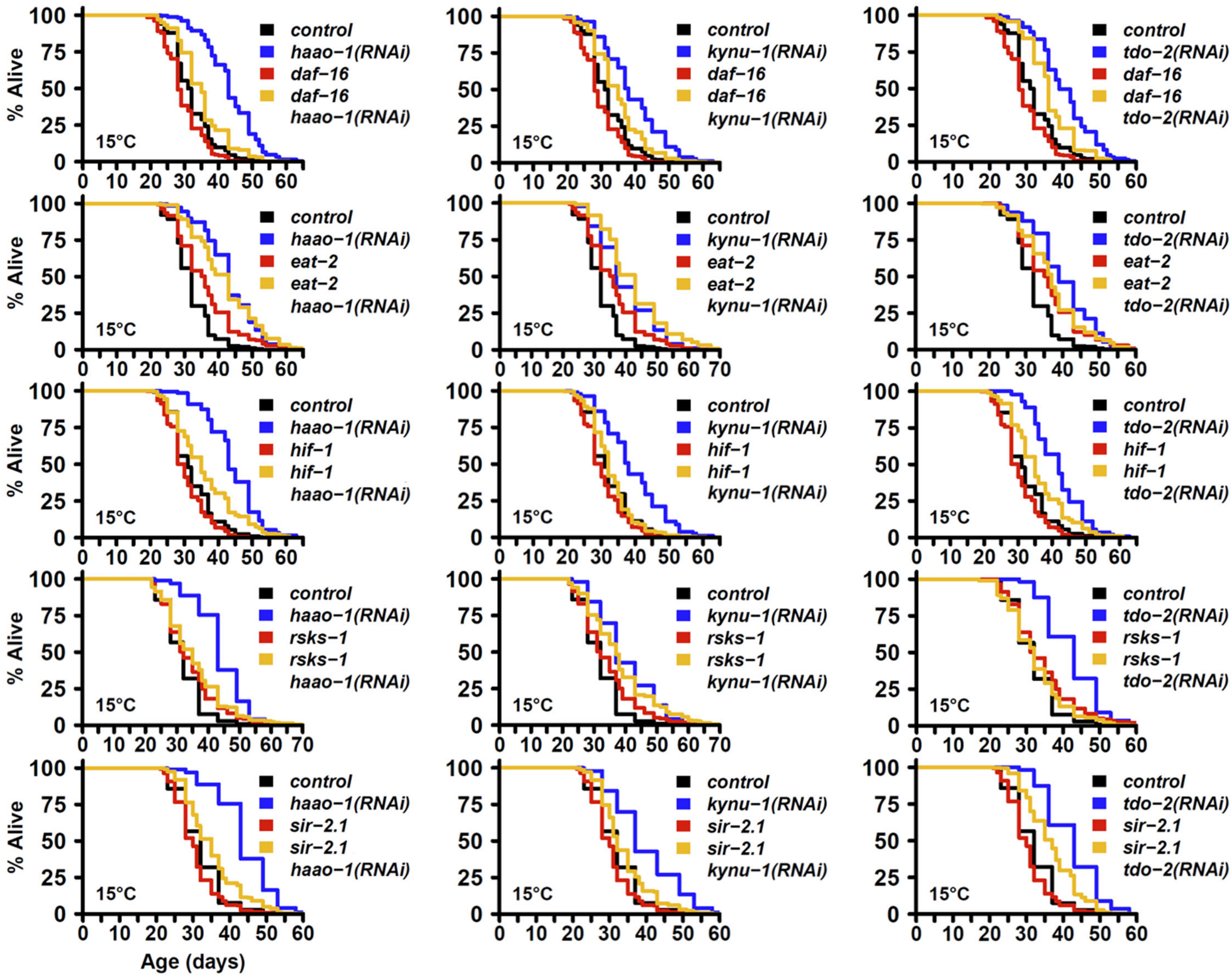

Supplementary Figure 3. *kynu-1*, *haao-1*, and *tdo-2* display distinct interaction patterns with established aging pathways at 15°C. Genetic interaction between *tdo-2*, *kynu-1*, and *haao-1* and established aging pathways. Each panel shows the impact on lifespan of RNAi knockdown of either *kynu-1*, *haao-1*, or *tdo-2* vs. *EV(RNAi)* in both wild type (N2) worms and worms with loss-of-function mutations in the following genes with a previous link to aging: *daf-16(mu86)* (strain CF1038), *eat-2(ad465)* (strain DA465), *hif-1(ia4)* (strain ZG31), *rsks-1(ok1255)* (strain RB1206), *sir-2.1(ok434)* (strain VC199). Summary statistics are provided in Supplementary Data 1 and source data are provided in Supplementary Data 2.

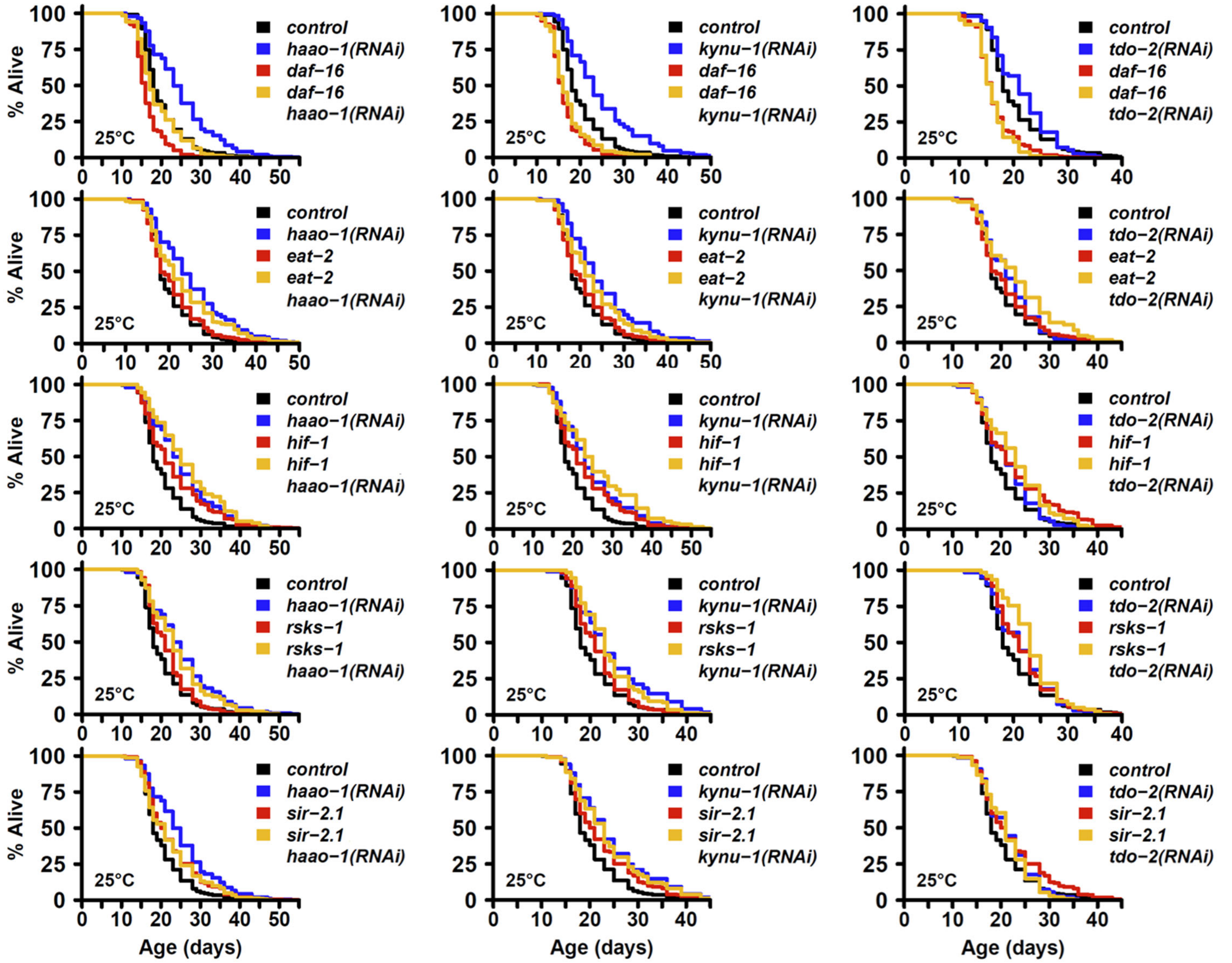

Supplementary Figure 4. *kynu-1*, *haao-1*, and *tdo-2* display distinct interaction patterns with established aging pathways at 25°C. Genetic interaction between *tdo-2*, *kynu-1*, and *haao-1* with established aging pathways. Each panel shows the impact on lifespan of RNAi knockdown of either *kynu-1*, *haao-1*, or *tdo-2* vs. *EV(RNAi)* in both wild type (N2) worms and worms with loss-of-function mutations in the following genes with a previous link to aging: *daf-16(mu86)* (strain CF1038), *eat-2(ad465)* (strain DA465), *hif-1(ia4)* (strain ZG31), *rsks-1(ok1255)* (strain RB1206), *sir-2.1(ok434)* (strain VC199). Summary statistics are provided in Supplementary Data 1 and source data are provided in Supplementary Data 2.

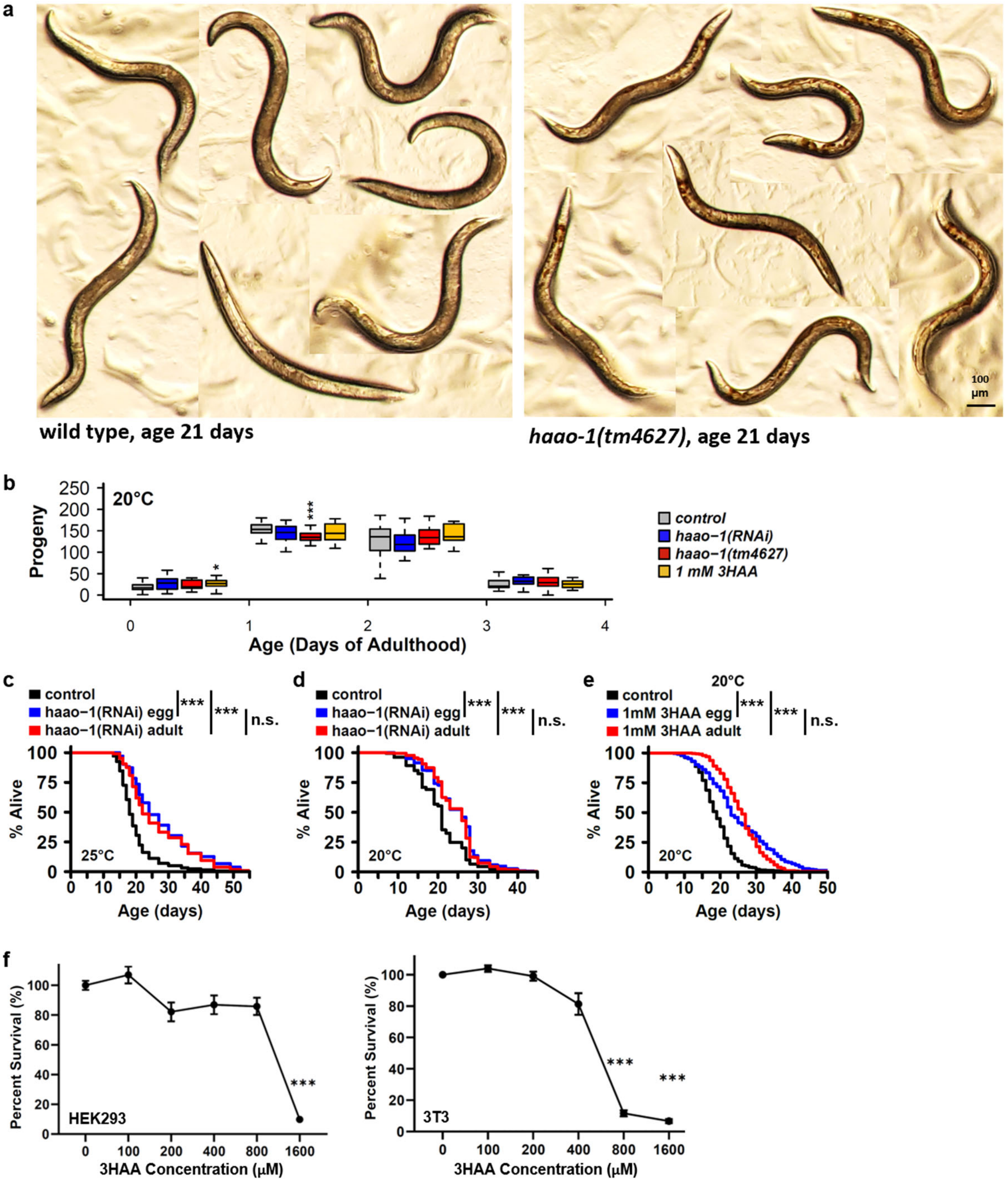

Supplementary Figure 5. 3HAA accumulates in animals with reduced *haao-1* and is sufficient to extend lifespan and metrics of health. (**a**) *haao-1(tm4627)* (bottom) *C. elegans* accumulate a red coloration as they age that is not present in wild type (top) animals (brightfield images; age 21 days). (**b**) 3HAA supplementation or *haao-1* largely does not affect brood size in *C. elegans* (with the exception of modest shifts during early reproduction). RNAi knockdown of *haao-1* extends lifespan to a similar degree when initiated at both egg and the first day of adulthood at both (**c**) 25°C and (**d**) 20°C. (**e**) 1 mM 3HAA extends lifespan to a similar degree when initiated at egg and the first day of adulthood at 20°C. (**f**) 3HAA cytotoxicity in HEK293 and 3T3 cell culture. * $p < 0.05$, ** $p < 0.01$, *** $p < 0.001$ vs. *EV(RNAi)* or 0 mM control (Welch's t test, panels b, f, g) or indicated comparison (log-rank test; panels c-e). Summary statistics are provided in Supplementary Data 1 and source data are provided in Supplementary Data 2.

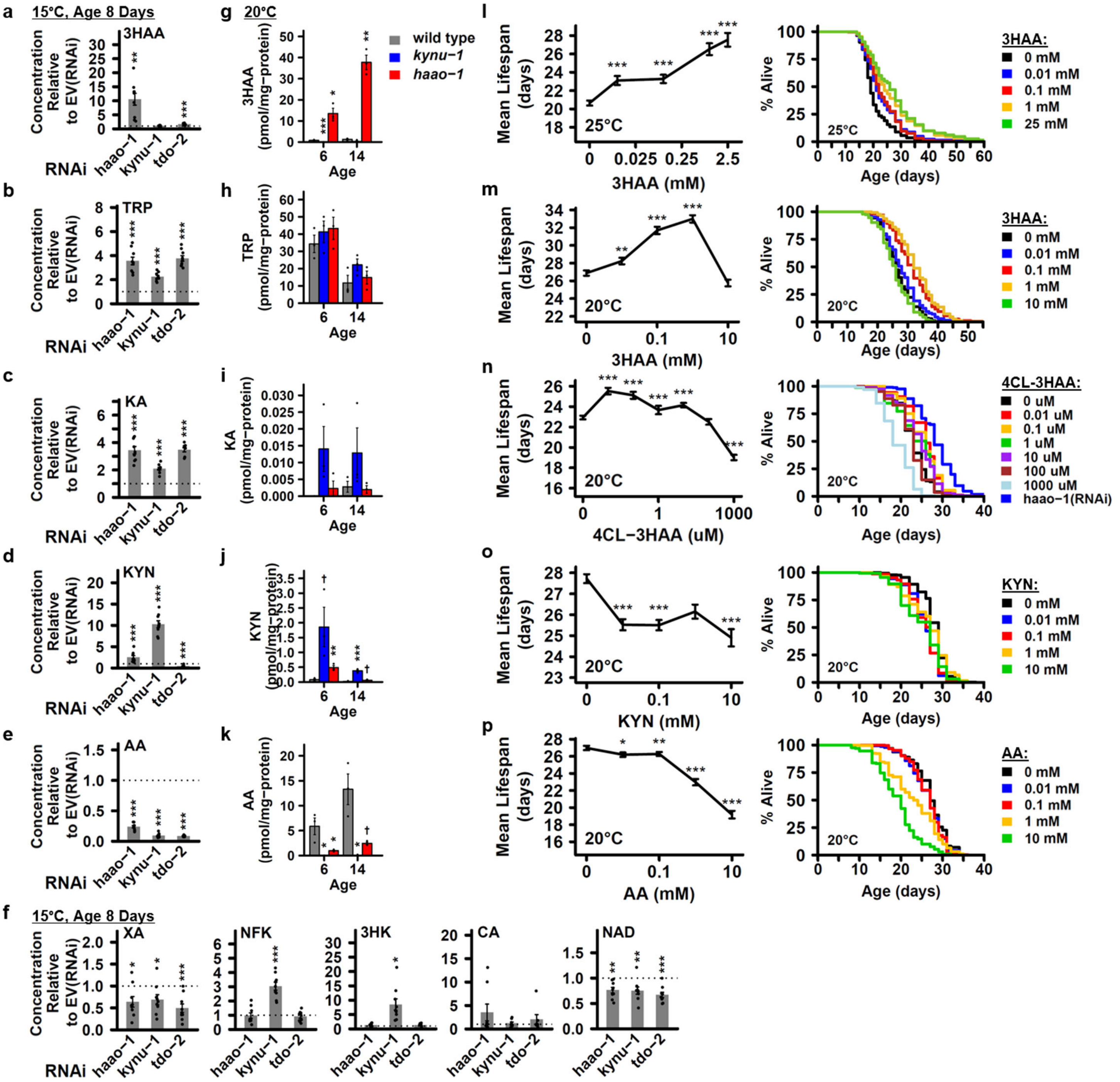

Supplementary Figure 6. The impact of kynurenine pathway metabolites on *C. elegans* lifespan. (**a-f**) RNAi knockdown of *kynu-1*, *haao-1*, or *tdo-2* produces distinct patterns of kynurenine metabolites relative to *EV(RNAi)* at 15°C at 8 days of age (LC-MS/MS). (**g-k**) *C. elegans* homozygous for the *kynu-1(tm4924) haao-1(tm4627)* deletion alleles have distinct patterns of kynurenine pathway metabolites relative to wild type at 20°C and 6 or 14 days of age (LC-MS/MS). (**l-p**) Supplementing *C. elegans* media with different kynurenine pathway metabolites produces distinct lifespan effects. Error bars indicate standard error of mean. * $p < 0.05$, ** $p < 0.01$, *** $p < 0.001$ compared to age-matched *EV(RNAi),* wild type, or 0 mM control (Welch's t test, panels a-k; log-rank test panels l-p). Summary statistics are provided in Supplementary Data 1 and source data are provided in Supplementary Data 2.

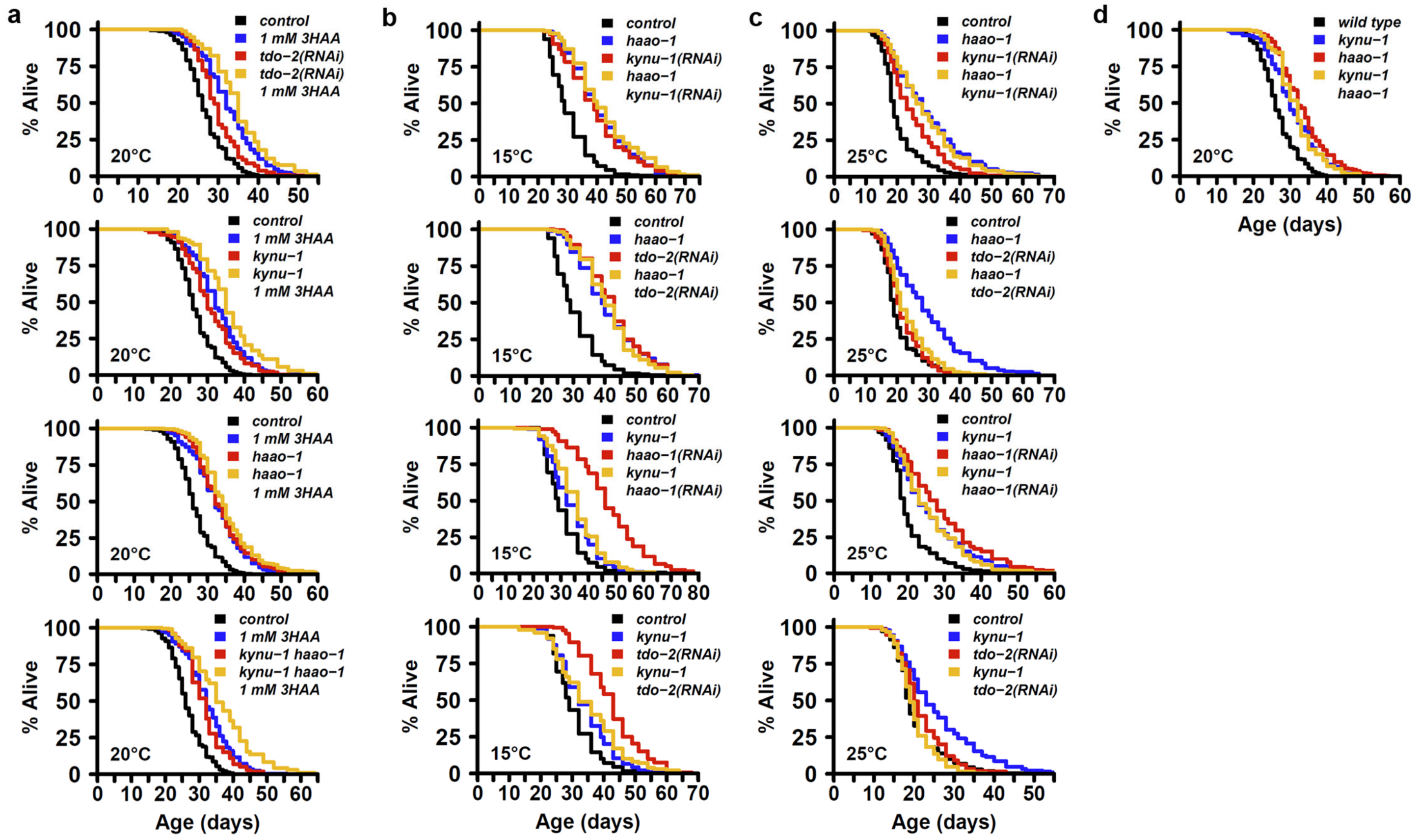

Supplementary Figure 7. Interaction between kynurenine pathway genes and 3HAA. (**a**) 1 mM 3HAA extends lifespan to a degree similar to wild type *C. elegans* in animals deficient for *tdo-2* (top) and *kynu-1* (middle top), but not *haao-1* (middle bottom). Knockout of *kynu-1* rescues the ability of 3HAA to extend lifespan in *haao-1* deficient animals (bottom bottom). Genetic interaction between *tdo-2*, *kynu-1*, and *haao-1* with respect to lifespan at (**b**) 15°C, (**c**) 25°C, and (**d**) 20°C. In all panels, *haao-1* refers to the *haao-1(tm4627)* knockout strain and *kynu-1* refers to the *kynu-1(4924)* knockout strain, while *tdo-2(RNAi)*, *kynu-1(RNAi)*, and *haao-1(RNAi)* refer to knockdown of the respective genes by RNAi. Summary statistics are provided in Supplementary Data 1 and source data are provided in Supplementary Data 2.

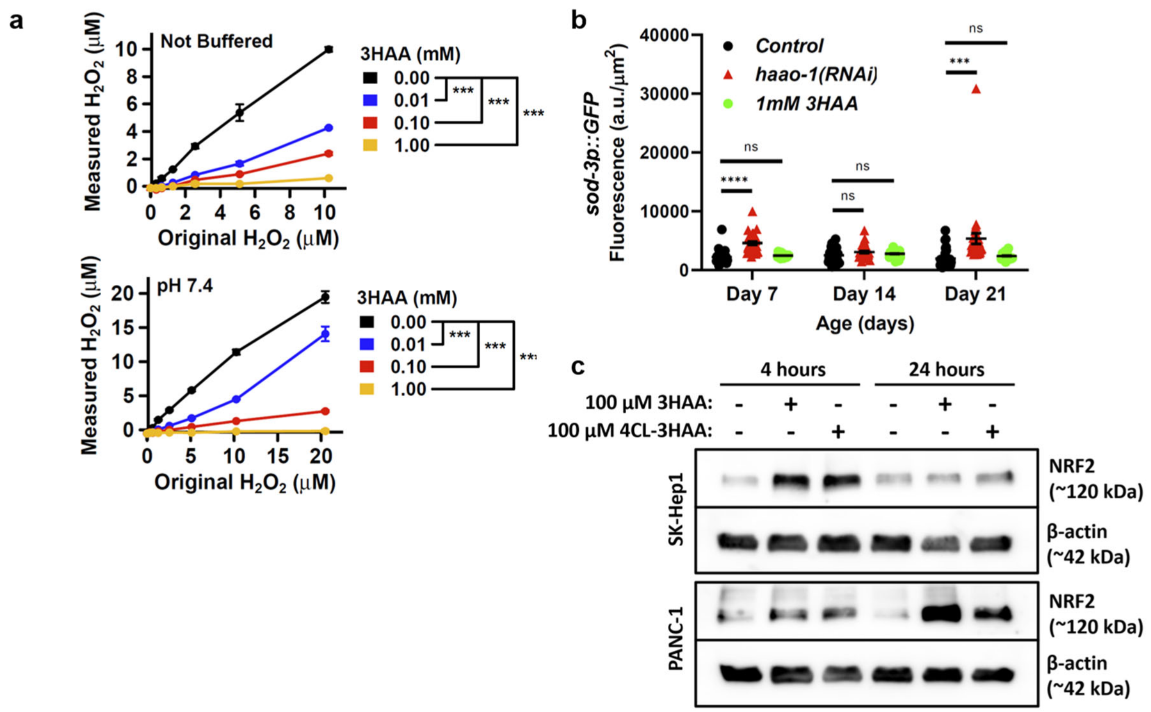

Supplementary Figure 8. 3HAA degrades hydrogen peroxide and activates oxidative stress response pathways in *C. elegans*. (**a**) 3HAA reduces detectable H_2O_2 in a dose-dependent manner in unbuffered (top) or buffered (bottom; pH 7.4) solution. (**b**) RNAi knockdown of *haao-1,* but not 1 mM 3HAA supplementation, mildly increases expression of a transgenically-expressed fluorescent DAF-16 transcriptional reporter (*sod-3p::GFP*) in worms fed *E. coli* strain HT115. (**c**) NRF2 is activated by 100 μM 3HAA in cultured human SK-Hep1 and PANC-1 cells (Western Blot). Error bars indicate standard error of mean. * $p < 0.05$, ** $p < 0.01$, *** $p < 0.001$ vs. 0 mM 3HAA (ANOVA; panel a) or age-matched control (t test; panels b, c). Summary statistics are provided in Supplementary Data 1 and source data are provided in Supplementary Data 2.

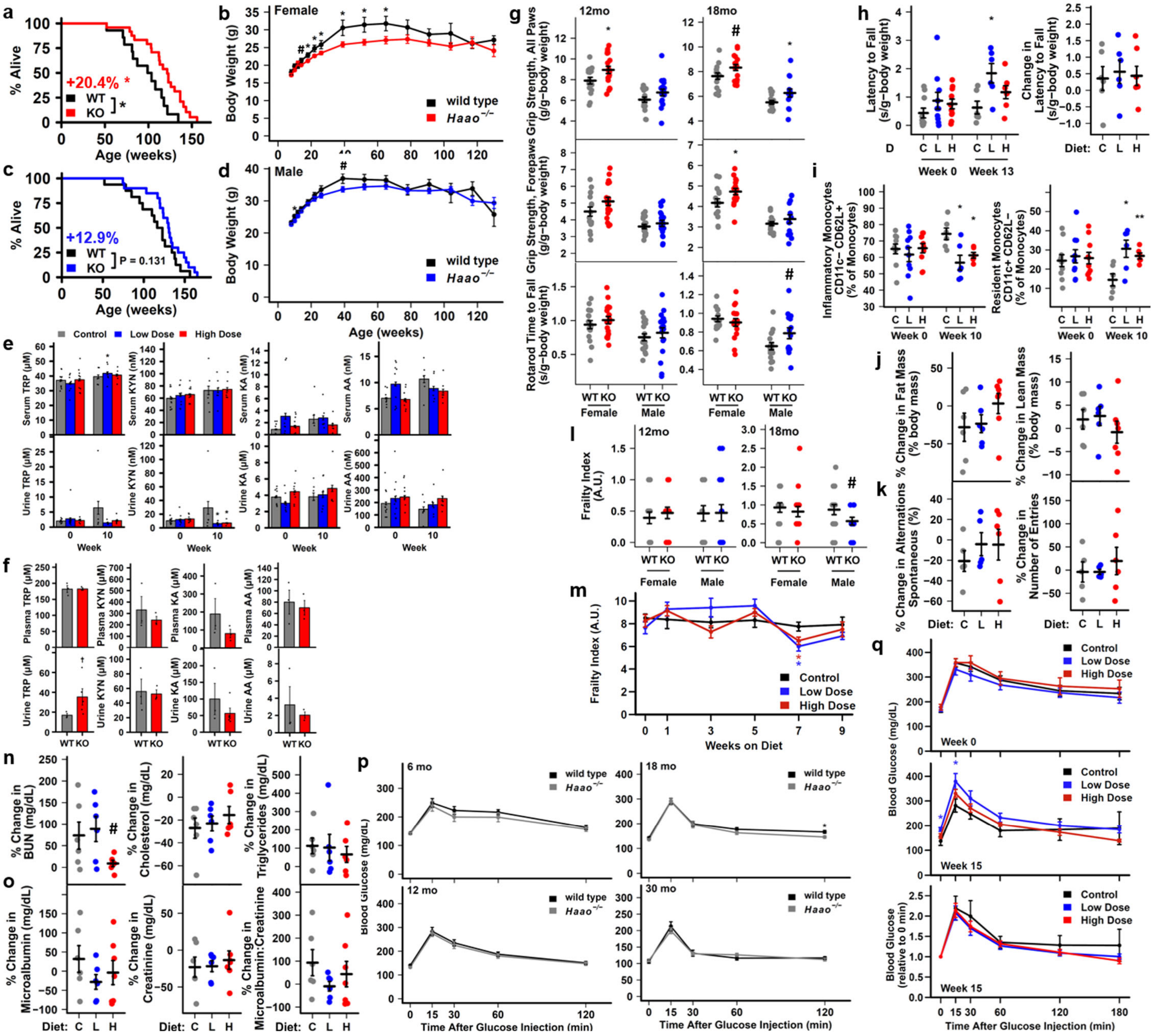

Supplementary Figure 9. Additional detail on the impact of elevating physiological 3HAA in mice. Deletion of *Haao* (*Haao*$^{-/-}$; KO) extends lifespan and reduces early- to mid-life body weight (**a, b**) significantly in female ($N_{WT} = 15$, $N_{KO} = 24$) but (**c, d**) only as a trend in male ($N_{WT} = 22$, $N_{KO} = 21$) C57BL/6N mice. Dietary 3HAA resulted in reduced urine, but not serum, KYN relative to control diet after 10 weeks, while TRP, KA, and AA were not altered in the serum or urine of (**e**) 27-month-old C57BL/6J mice fed chow supplemented with 3HAA or (**f**) the plasma or urine of *Haao*$^{-/-}$ mice. (**g**) *Haao*$^{-/-}$ mice tend toward increased grip strength and rotarod performance at 12 and 18 months of age. (**h**) Mice fed low dose 3HAA diet have increased rotarod performance after 13 weeks relative to mice fed a control diet (left); however, this may result from survivor bias, as individual change in performance for mice surviving to 13 weeks is not changed (right). (**i**) Mice fed 3HAA diet had fewer inflammatory monocytes and more resident monocytes as a percentage of total monocytes relative to mice fed control diet after 10 weeks. Other measured parameters were not impacted by 3HAA diet or

Haao deletion include (**j**) body composition, (**k**) Y-maze performance, (**l, m**) frailty index (FI), (**n**) blood urea nitrogen (BUN; left), cholesterol (center), or triglycerides (right), (**o**) urine microalbumin (A; left), creatinine (B; center), or A:C ratio (right), and (**p, q**) glucose tolerance. Impaired glucose response in mice fed low dose 3HAA diet (panel q, center) appears to be driven by elevated fasting glucose (panel q, bottom). $^{\#}$ $p < 0.1$, †,* $p < 0.05$, **, ‡ $p < 0.01$, *** $p < 0.001$ vs. control diet (log rank test, panels a, c; Welch's t test, panels b-p). For panel e, *, **, *** indicate significance vs. Week 0; †, ‡ indicate significance vs. control diet. Summary statistics are provided in Supplementary Data 1 and source data are provided in Supplementary Data 2.

a

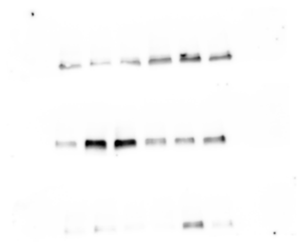

b

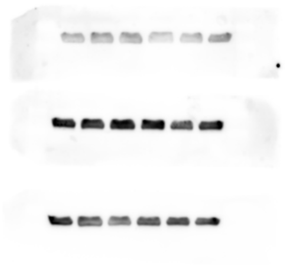

c

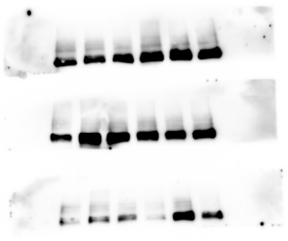

d

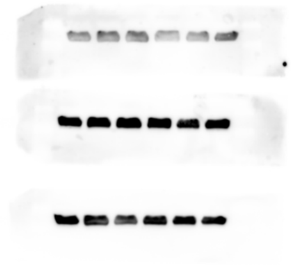

Supplementary Figure 10. Western blot raw images for NRF2 western blots. Uncropped raw images of the stained membrane for the Western blot presented in Supplementary Figure 8 are provided. Images show the original images for antibodies targeting (a) NRF2 in SK-Hep1 cells (middle band), (b) β-actin in SK-Hep1 cells (middle band), (c) NRF2 in PANC-1 cells (bottom band), and (d) β-actin in PANC-1 cells (bottom band).
