# Supplementary material for: On the benefits of the tryptophan metabolite 3-hydroxyanthranilic acid in Caenorhabditis elegans and mouse aging
Source: Nat Commun. 2023 Dec 14;14:8338. doi: 10.1038/s41467-023-43527-1 (PMC10721613; doi:10.1038/s41467-023-43527-1)
Supplement: Supplementary file 3 — Description of Additional Supplementary Files [file 41467_2023_43527_MOESM3_ESM.pdf]

Description of Additional Supplementary Files

Supplementary Data 1: Contains summary statistics for all experiments outlined in the Results, Figures, and Supplementary Figures, RNAi clones used in this work, and Caenorhabditis elegans strains used in this work.
