# Supplementary material for: On the benefits of the tryptophan metabolite 3-hydroxyanthranilic acid in Caenorhabditis elegans and mouse aging
Source: Nat Commun. 2023 Dec 14;14:8338. doi: 10.1038/s41467-023-43527-1 (PMC10721613; doi:10.1038/s41467-023-43527-1)
Supplement: Supplementary file 5 — Reporting Summary [file 41467_2023_43527_MOESM5_ESM.pdf]

nature portfolio

Corresponding author(s): George Sutphin

Last updated by author(s): Nov 1, 2023

Reporting Summary

Nature Portfolio wishes to improve the reproducibility of the work that we publish. This form provides structure for consistency and transparency in reporting. For further information on Nature Portfolio policies, see our Editorial Policies and the Editorial Policy Checklist.

Statistics

For all statistical analyses, confirm that the following items are present in the figure legend, table legend, main text, or Methods section.

n/a	Confirmed	
☐	☒	The exact sample size (*n*) for each experimental group/condition, given as a discrete number and unit of measurement
☐	☒	A statement on whether measurements were taken from distinct samples or whether the same sample was measured repeatedly
☐	☒	The statistical test(s) used AND whether they are one- or two-sided<br>*Only common tests should be described solely by name; describe more complex techniques in the Methods section.*
☐	☒	A description of all covariates tested
☐	☒	A description of any assumptions or corrections, such as tests of normality and adjustment for multiple comparisons
☐	☒	A full description of the statistical parameters including central tendency (e.g. means) or other basic estimates (e.g. regression coefficient) AND variation (e.g. standard deviation) or associated estimates of uncertainty (e.g. confidence intervals)
☐	☒	For null hypothesis testing, the test statistic (e.g. *F*, *t*, *r*) with confidence intervals, effect sizes, degrees of freedom and *P* value noted<br>*Give P values as exact values whenever suitable.*
☒	☐	For Bayesian analysis, information on the choice of priors and Markov chain Monte Carlo settings
☒	☐	For hierarchical and complex designs, identification of the appropriate level for tests and full reporting of outcomes
☒	☐	Estimates of effect sizes (e.g. Cohen's *d*, Pearson's *r*), indicating how they were calculated

Our web collection on statistics for biologists contains articles on many of the points above.

Software and code

Policy information about availability of computer code

Data collection	No code was used to collect the data.
Data analysis	The majority of the analysis was conducted using publicly available packages of R 4.3.0. A small subset of analyses were conducted Graph Pad Prism (version 8.4.3.686). Fluorescence of transgenic C.elegans strains was conducted using a custom script called LightSaver, which is available on GitHub. We quantified fluorescence using a tool that we developed called LightSaver (version 0.1), which is available on GitHub. All of these details are included in our Methods section, with appropriate references.

For manuscripts utilizing custom algorithms or software that are central to the research but not yet described in published literature, software must be made available to editors and reviewers. We strongly encourage code deposition in a community repository (e.g. GitHub). See the Nature Portfolio guidelines for submitting code & software for further information.

Data

Policy information about availability of data

All manuscripts must include a data availability statement. This statement should provide the following information, where applicable:

- Accession codes, unique identifiers, or web links for publicly available datasets
- A description of any restrictions on data availability
- For clinical datasets or third party data, please ensure that the statement adheres to our policy

The datasets generated and/or analyzed during the current study are available as Supplementary Data 1.

Research involving human participants, their data, or biological material

Policy information about studies with human participants or human data. See also policy information about sex, gender (identity/presentation), and sexual orientation and race, ethnicity and racism.

Reporting on sex and gender	No human data.
Reporting on race, ethnicity, or other socially relevant groupings	No human data.
Population characteristics	No human data.
Recruitment	No human data.
Ethics oversight	No human data.

Note that full information on the approval of the study protocol must also be provided in the manuscript.

Field-specific reporting

Please select the one below that is the best fit for your research. If you are not sure, read the appropriate sections before making your selection.

- [x] Life sciences
- [ ] Behavioural & social sciences
- [ ] Ecological, evolutionary & environmental sciences

For a reference copy of the document with all sections, see nature.com/documents/nr-reporting-summary-flat.pdf

Life sciences study design

All studies must disclose on these points even when the disclosure is negative.

Sample size	For C. elegans experiments, we follow the standard practice of including ~100 animals per test group for each lifespan study, and repeating our work in minimum duplicate (usually triplicate). A sample size of 100 animals provides 80% power to detect a 10% change in lifespan, and 95% power to detect a 20% change in lifespan (PMID28713422). The two included mouse studies were designed as pilot studies, and were therefore known to be underpowered to detect differences. For the 3HAA diet study, we included all available mice (only males). For the HAAO knockout mouse study we bred mice and included individuals until we reached a minimal group size of 15 per sex per genotype (wild type and homozygous Haao knockout). This study was designed as a pilot study. Sample size was not selected to optimize power; however, we added mice to a minimum of 15 mice/sex/group, which corresponds to 75% power to detect a 20% change in lifespan in the combined group, and 50% power to detect a 20% change in lifespan in each sex. Both were designed as pilot studies; sample size was not determined.
Data exclusions	Data were excluded for C. elegans studies only if the worms fled the plate (in which case they are not recorded in the study), following standard practice. For mouse studies, we censored mice that died of causes unrelated to aging. This is usually young male mice that die from fighting, but also includes incidental deaths from flooded cages or similar situations.
Replication	For worm experiments, most experiments were repeated in triplicate or greater, with a few only conducted in duplicate. While most observations replicated, for experiments in which the outcome was not consistent between 2 to 3 replicates, at least one additional replicate was added to resolve the outcome. The mouse studies were intended as pilot studies, and extremely resource limited. We have not replicated these studies yet, but intend to do so once we have sufficient funding. The strength of our interpretation explicitly takes the replication issue into account for the mouse studies.
Randomization	C. elegans experimental animal are all randomly selected from the same populations where possible (or parallel populations grown on the same batch of plates for comparison between different strains). All strains are periodically thawed from frozen stocks and passaged for several generation on well-fed plates before experiments are conducted. Mice in the 3HAA diet study were randomly assigned to each diet by cage. Mice in the Haao knockout study were bred from heterozygous parents. All surviving wild type and homozygous knockout mice were selected from litters during the breeding period following genotyping (i.e., random selection of animals from each genotype from the same litters).
Blinding	For C. elegans studies, individuals scoring experiments were blinded to the groups by a second individual, and not unblinded until the study was complete. For mouse studies, mice fed 3HAA and Haao knockout mice are not visually or behaviorally distinguishable from their respective controls. Technicians working with the mice were blinded to the test groups while experiments were conducted.

Reporting for specific materials, systems and methods

We require information from authors about some types of materials, experimental systems and methods used in many studies. Here, indicate whether each material, system or method listed is relevant to your study. If you are not sure if a list item applies to your research, read the appropriate section before selecting a response.

Materials & experimental systems

n/a	Involved in the study
[ ]	[x] Antibodies
[ ]	[x] Eukaryotic cell lines
[x]	[ ] Palaeontology and archaeology
[ ]	[x] Animals and other organisms
[x]	[ ] Clinical data
[x]	[ ] Dual use research of concern
[x]	[ ] Plants

Methods

n/a	Involved in the study
[x]	[ ] ChIP-seq
[ ]	[x] Flow cytometry
[x]	[ ] MRI-based neuroimaging

Antibodies

Antibodies used

The following antibodies were used: anti-Nrf2 (primary; Abcam ab62352, clone EP1808Y), anti-β-actin (primary; Cell Signaling Technology 3700S, clone 8H10D10), goat anti-mouse IgG2b heavy chain (HRP) preabsorbed (secondary; Abcam ab98703, polyclonal) and goat anti-rabbit IgG2b heavy chain (HRP) preabsorbed (secondary; Santa Cruz Biotechnology sc-2004, polyclonal). Primary antibodies were used at a 1:1000 dilution. Secondary antibodies were used at a 1:3000 dilution.

Validation

Abcam ab62352 was validated for Western blot by the manufacturer in human NRF2 knockout cells and has been used in more than 400 publications (e.g., PMID34369898). CST 3700S was validated for Western blot by the manufacturer across many mammalian species, including multiple human cell types, and has been used used in more than 4000 publications (e.g., PMID37106272). Abcam ab98703 was validated by the manufacturer for Western blot in human and rat cells and has been used in 2 publications (PMID36222485, PMID32759326). Santa Cruz Biotechnology sc-2004 was validated for Western blot by the manufacturer in multiple human cell lines and has been used in more than 1000 publications (e.g., PMID29955039).

Eukaryotic cell lines

Policy information about cell lines and Sex and Gender in Research

Cell line source(s)

PANC-1 and SK-Hep1 were both obtained from the University of Arizona Cancer Experimental Mouse Shared Resource.

Authentication

Cell lines were authenticated by the University of Arizona Cancer Experimental Mouse Shared Resource by morphology and STR profiling.

Mycoplasma contamination

All cell lines tested negative for mycoplasma

Commonly misidentified lines
(See ICLAC register)

No commonly misidentified lines were used in this work.

Animals and other research organisms

Policy information about studies involving animals; ARRIVE guidelines recommended for reporting animal research, and Sex and Gender in Research

Laboratory animals

The 3HAA diet mouse study used C57B6L/6J mice. Mice were obtained from The Jackson Laboratory at 27 months of age and monitored for remaining lifespan. Additional phenotyping was conducted at 27 months of age, and between 30 and 32 months of age. The Haao knockout study was conducted in the C57BL/6N background bearing the Haaotm1b(KOMP)Mbp allele (knockout line provided by the international mouse phenotyping consortium). Mice were bred in house at the University of Arizona and monitored throughout life to determine lifespan Phenotyping was conducted for subsets of mice at 4, 6, 12, 18, and 30 months of age. Specific ages for each experiment are noted in the Results and Methods section.

The following strains were obtained from the Caenorhabditis Genetic Center (CGC) in the College of Biological Sciences at the University of Minnesota: daf 16(mu86) I (CF1038), eat 2(ad465) II (DA465), hif 1(ia4) V (ZG31), rsks 1(ok1255) III (RB1206), sir 2.1 (ok434) IV (VC199), rrf-1(pk1417) I (MAH23)53, rde-1(ne219) V; kzIs9[(pKK1260) lin-26p::NLS::GFP + (pKK1253) lin-26p::rde-1 + rol-6 (su1006)] (NR222), lin 15B(n744) X; uIs57[unc-119p::YFP + unc-119p::sid-1 + mec-6p::mec-6] (TU3335), rde-1(ne219) V; kbIs7 [nhx-2p::rde-1 + rol-6(su1006)] (VP303), rde-1(ne300) V; neIs9[myo-3::HA::RDE-1 + rol-6(su1006)] X (WM118), wgIs341 [skn-1::TY1::EGFP::3xFLAG + unc-119(+)] (OP341), dvIs19[(pAF15)gst-4p::GFP::NLS] (CL2166), ldIs3[gcs-1p::GFP + rol-6(su1006)] (LD1171)54, muIs84[(pAD76) sod-3p::GFP + rol-6(su1006)] (CF1553), skn-1(zj15) IV (QV225), and jrIs1[rpl-17p::HyPer + unc-119(+)] (JV1)17 . Strains kynu-1(tm4924) X (FX04924; backcrossed 6x to N2 to create strain GLS129) and haao-1(tm4627) V (FX04627; backcrossed 6x to N2 to create strain GLS130) were obtained from the C. elegans National Bioresource Project (NBRP) at the School of Medicine at the Tokyo Women's Medical University. Wild type (N2) worms were originally obtained from Dr. Matt Kaeberlein (University of Washington, Seattle, WA, USA). Strain RB1206 was generated by the C. elegans Gene Knockout Project at the Oklahoma Medical Research Foundation as part of the International C. elegans Gene Knockout Consortium55. Strain VC199 was generated by the C. elegans Reverse Genetics Core Facility at the University of British Columbia as part of the International C. elegans Gene Knockout Consortium55. Strain OP341 was constructed as part of the Regulatory Element Project, part of modENCODE56. Strain TU3335 was created by Calixto et al57. Strain VP303 was created by Espelt et al58. Strain LD1171 was created by Wang et al54. Strain haao-1(tm4627) V kynu-1(tm4924) X (GLS147) was generated by crossing GLS129 to GLS130. Strains haao-1 (syb2665)::wrmscarlet (PHX2665) and kynu-1(syb2691)::wrmscarlet (PHX2691) were created for this work using CRISPR/Cas9 precise gene insertion by SunyBiotech Corporation. All worms were bred in house at the University of Arizona. For lifespan studies, animals

	were monitored throughout life to determine lifespan. For other experiments, phenotypes were measured between day 1 and day 21 of adulthood. Specific ages for each experiment are noted in the Results and Methods sections.
Wild animals	None
Reporting on sex	The 3HAA mouse diet study was only conducted in male mice, as these were the only aged animals available and the study was intended as a pilot study for the impact of 3HAA on lifespan. The Haao knockout study was conducted in both male and female mice. Our analyses considered the population as a whole, as well as independent analyses of male and female mice.
Field-collected samples	None
Ethics oversight	The Jackson Laboratory and University of Arizona IACUC had oversight and approved of the 3HAA diet mouse study and Haao KO study, respectively

Note that full information on the approval of the study protocol must also be provided in the manuscript.

Flow Cytometry

Plots

Confirm that:

- [x] The axis labels state the marker and fluorochrome used (e.g. CD4-FITC).
- [x] The axis scales are clearly visible. Include numbers along axes only for bottom left plot of group (a 'group' is an analysis of identical markers).
- [x] All plots are contour plots with outliers or pseudocolor plots.
- [x] A numerical value for number of cells or percentage (with statistics) is provided.

Methodology

Sample preparation	Published in Ackert-Bicknell, C. L. et al. Aging Research Using Mouse Models. Curr. Protoc. Mouse Biol. 5, 95–133 (2015).
Instrument	Published in Ackert-Bicknell, C. L. et al. Aging Research Using Mouse Models. Curr. Protoc. Mouse Biol. 5, 95–133 (2015).
Software	Published in Ackert-Bicknell, C. L. et al. Aging Research Using Mouse Models. Curr. Protoc. Mouse Biol. 5, 95–133 (2015).
Cell population abundance	Published in Ackert-Bicknell, C. L. et al. Aging Research Using Mouse Models. Curr. Protoc. Mouse Biol. 5, 95–133 (2015).
Gating strategy	Published in Ackert-Bicknell, C. L. et al. Aging Research Using Mouse Models. Curr. Protoc. Mouse Biol. 5, 95–133 (2015).

- [x] Tick this box to confirm that a figure exemplifying the gating strategy is provided in the Supplementary Information.
